# Supplementary material for: Investigation of Rare Non-Coding Variants in Familial Multiple Myeloma
Source: Cells. 2022 Dec 26;12(1):96. doi: 10.3390/cells12010096 (PMC9818386; doi:10.3390/cells12010096)
Supplement: Supplementary file 1 [file cells-12-00096-s001.zip › supplementary figures_newresolute.pdf]

Supplementary Figures

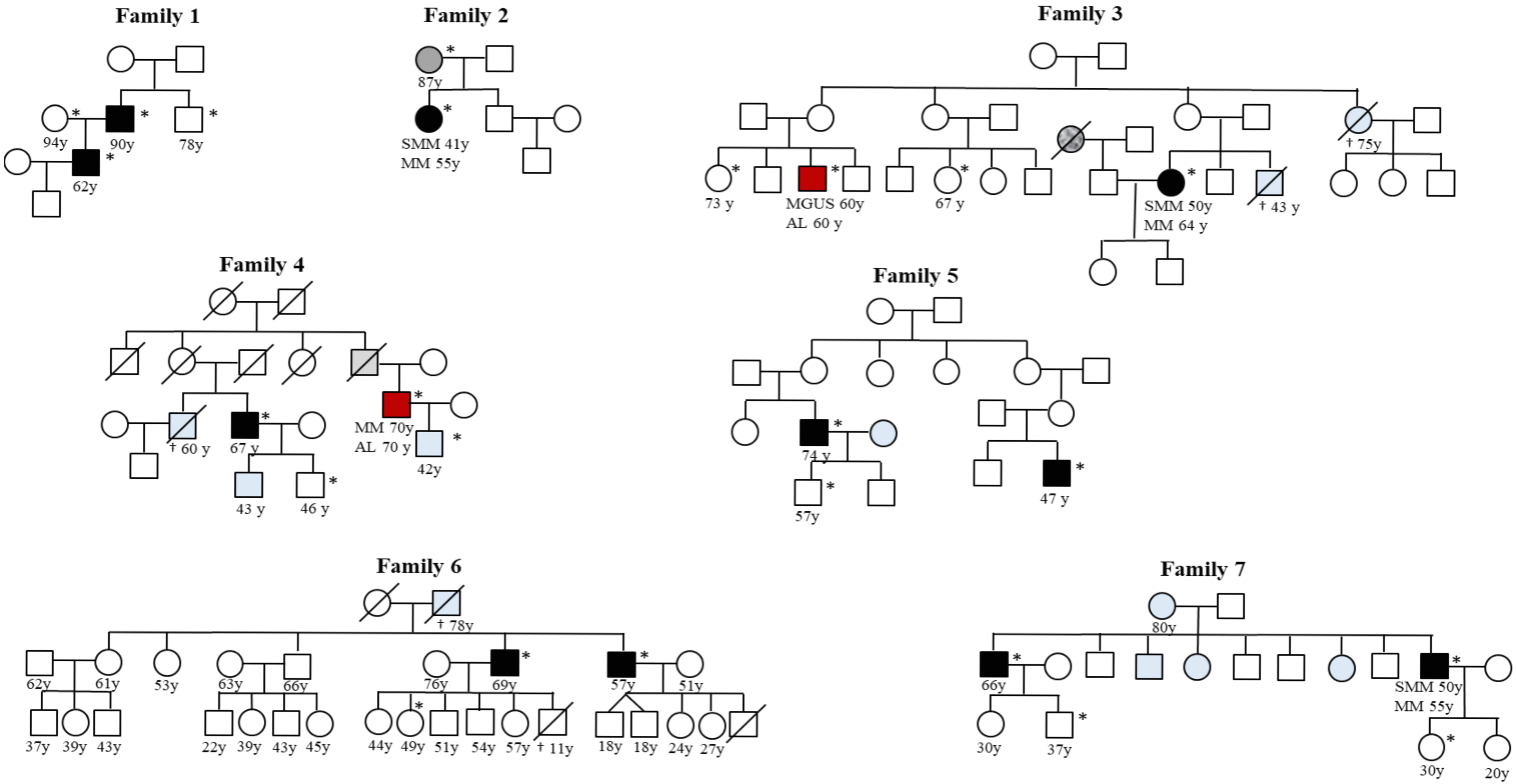
Family 1
94y
90y
78y
62y
Family 2
87y
SMM 41y
MM 55y
Family 3
73 y
MGUS 60y
AL 60 y
67 y
SMM 50y
MM 64 y
† 43 y
† 75y
Family 4
† 60 y
67 y
MM 70y
AL 70 y
42y
43 y
46 y
Family 5
74 y
57y
47 y
Family 6
† 78y
62y
61y
53y
63y
66y
76y
69y
57y
51y
37y
39y
43y
22y
39y
43y
45y
44y
49y
51y
54y
57y
† 11y
18y
18y
24y
27y
Family 7
80y
66y
SMM 50y
MM 55y
30y
37y
30y
20y

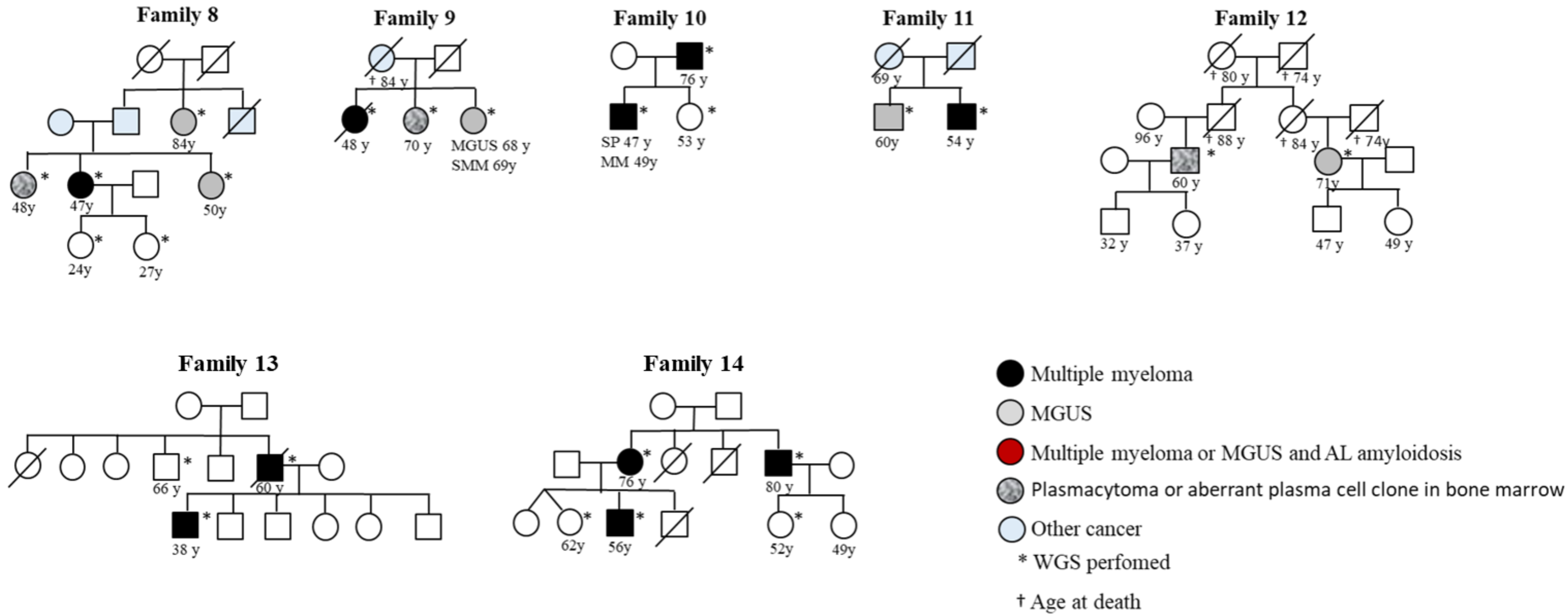

Supplementary Figure S1. Family pedigrees of the 14 families investigated in the study.

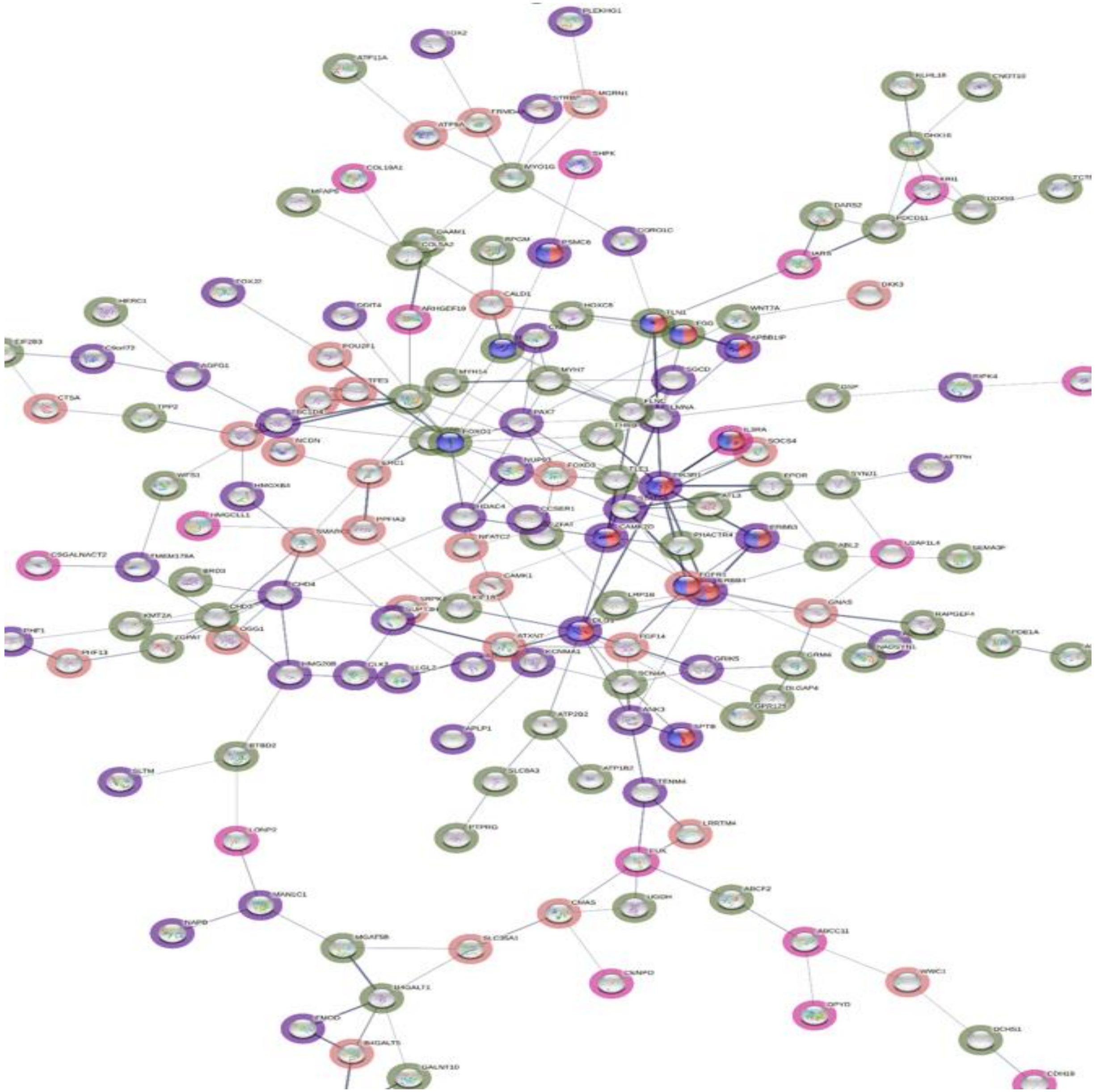

Supplementary Figure S2. Protein interaction network of a combination of genes with 5' and 3' UTR variants and genes with missense and loss-of-function variants generated by STRING. Proteins surrounded by green halo are missense and those surrounded by magenta are loss-of-function (LoF) variant related proteins. 5'UTR and 3'UTR variant proteins are surrounded by indigo and light red haloes respectively

Supplementary Figures S3–S21: UCSC plots of top 5‘ UTR and MAPK pathway variants

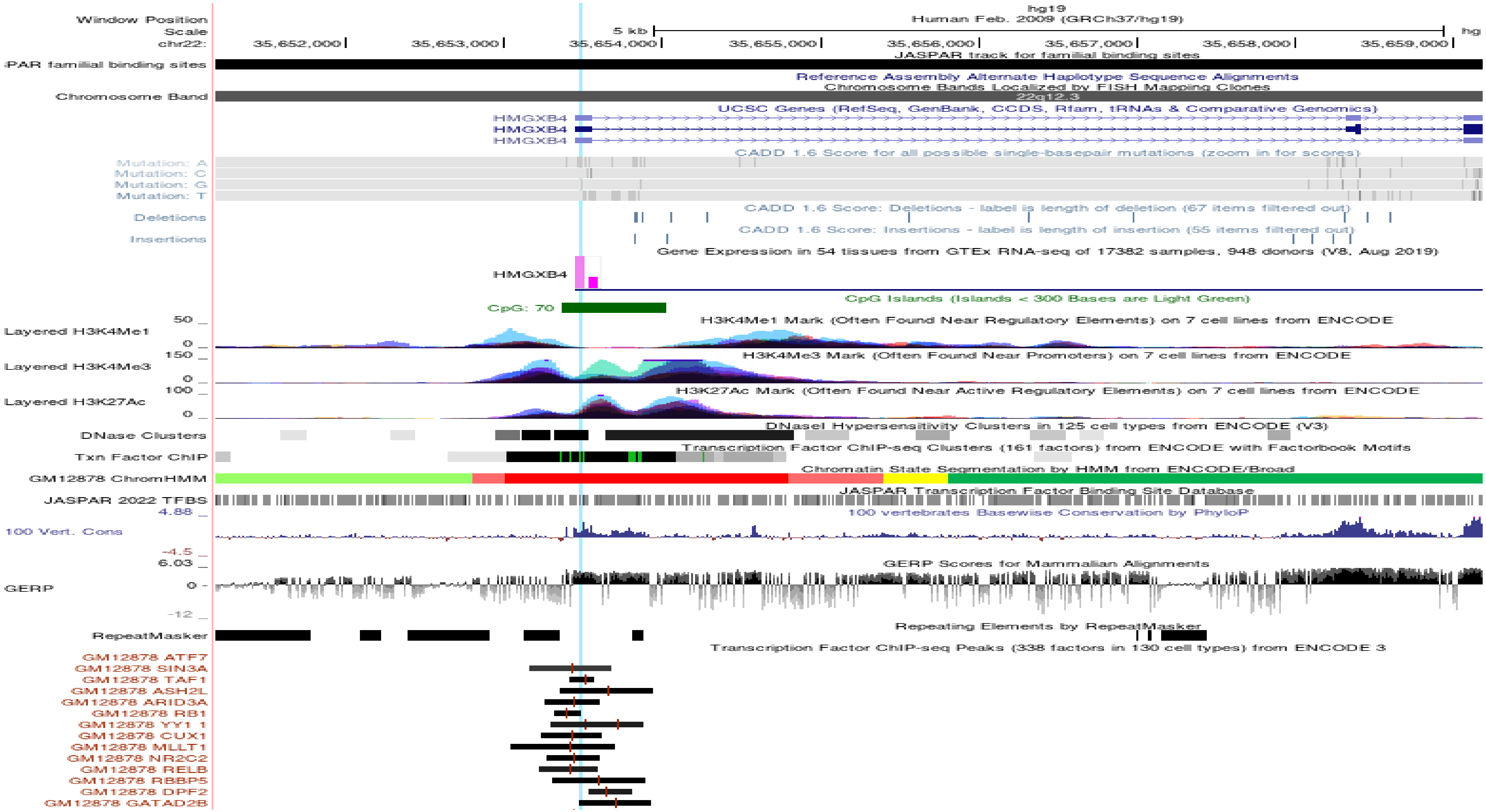

Supplementary Figure S3 *HMGXB4:22_35653479_C_A*

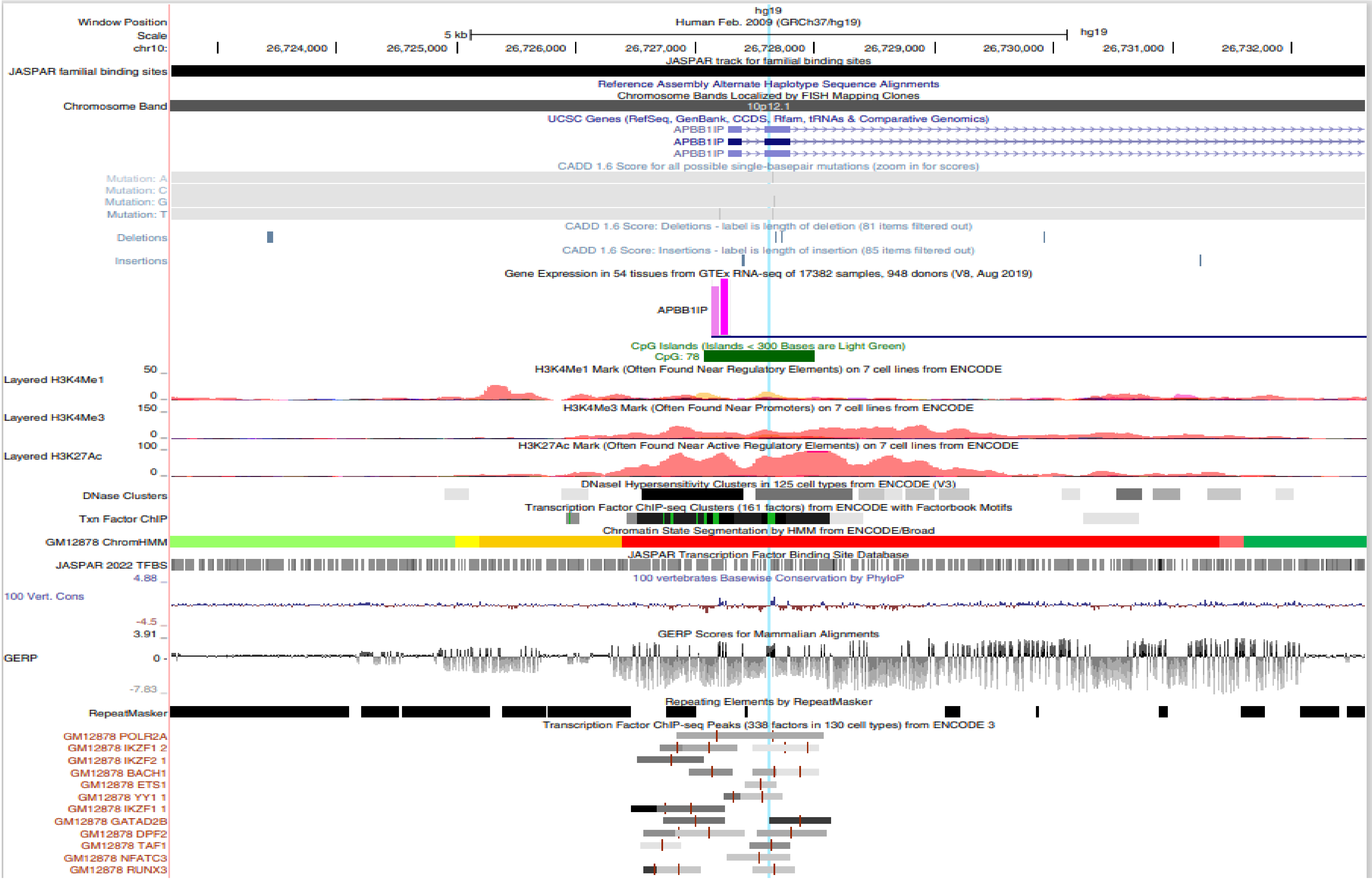

Supplementary Figure S4. *APBB1IP:10_26727608_C_G*

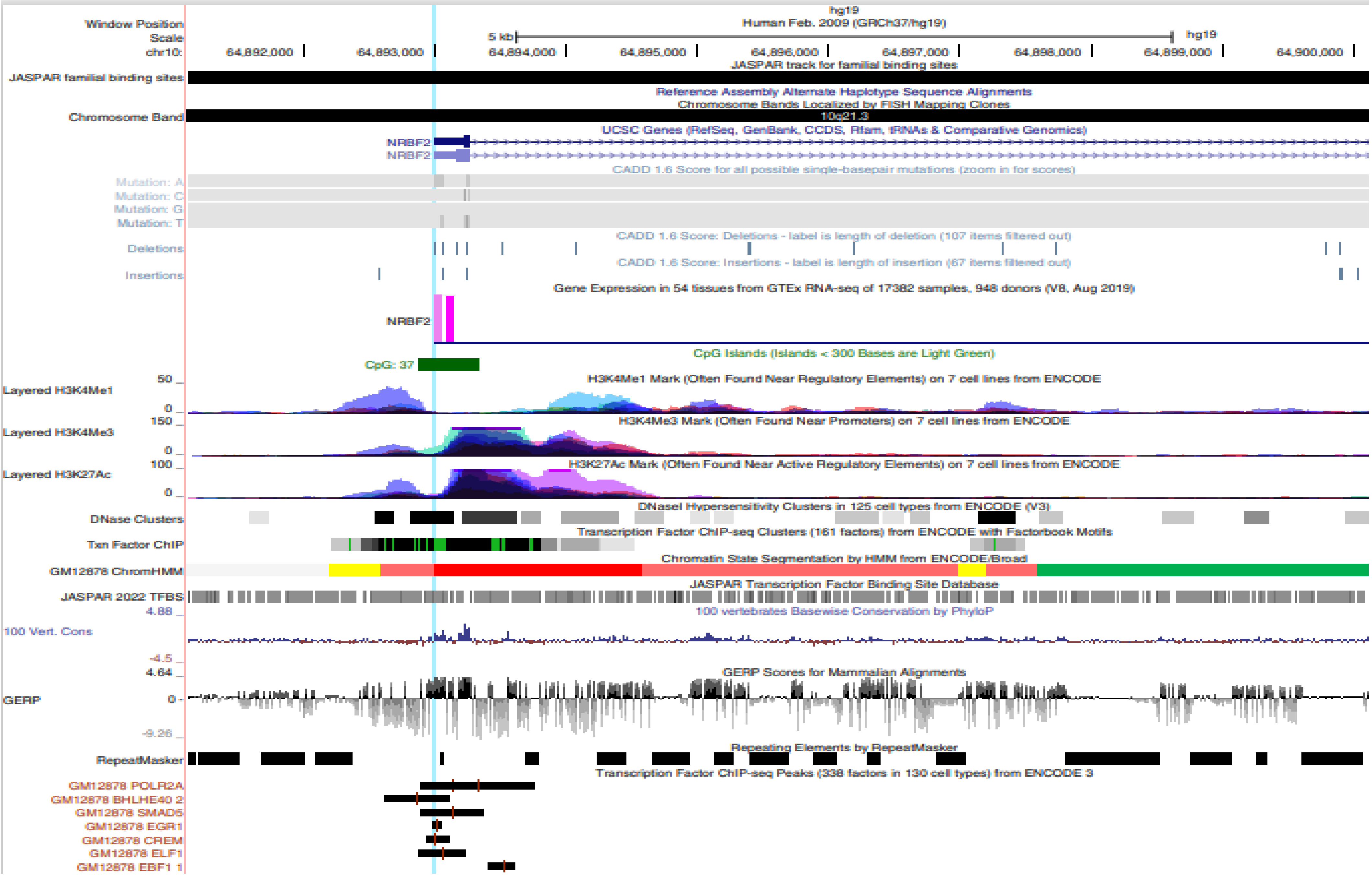

Supplementary Figure S5. *NRBF2*: 10_64893005_T_C

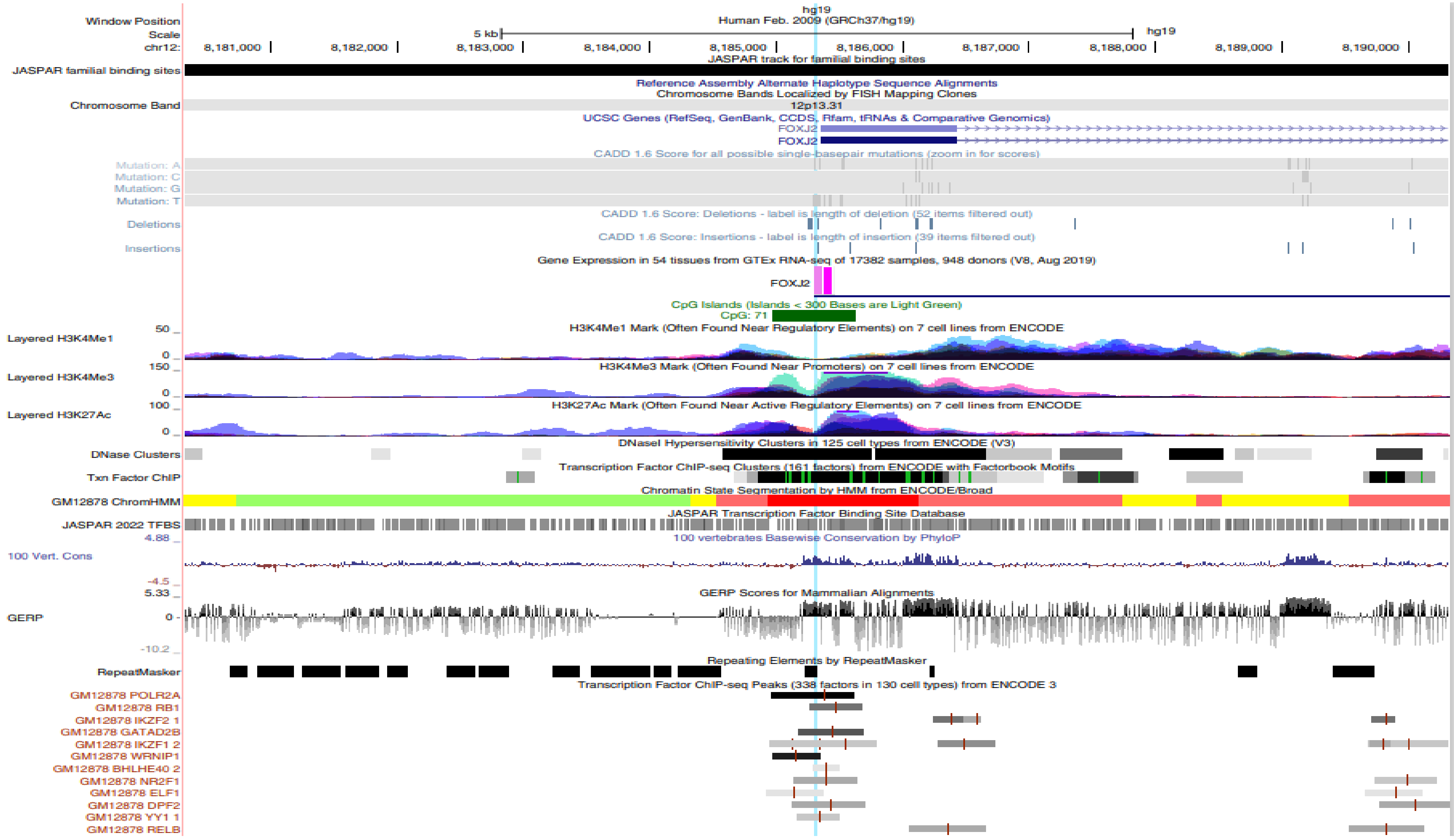

Supplementary Figure S6. *FOXJ2:12_8185317_GGAGCC_G*

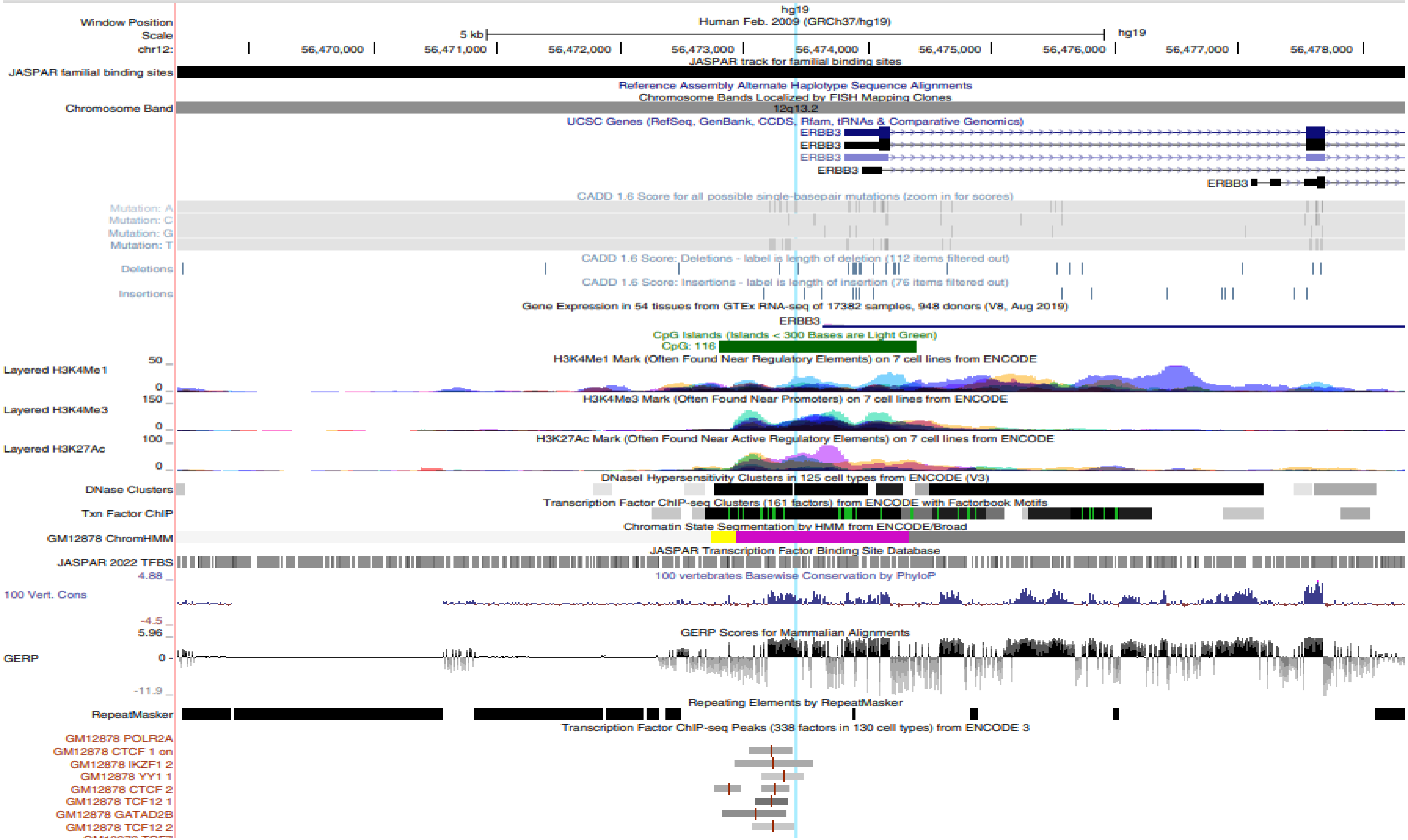

Supplementary Figure S7. *ERBB3:12_56473408_C_T*

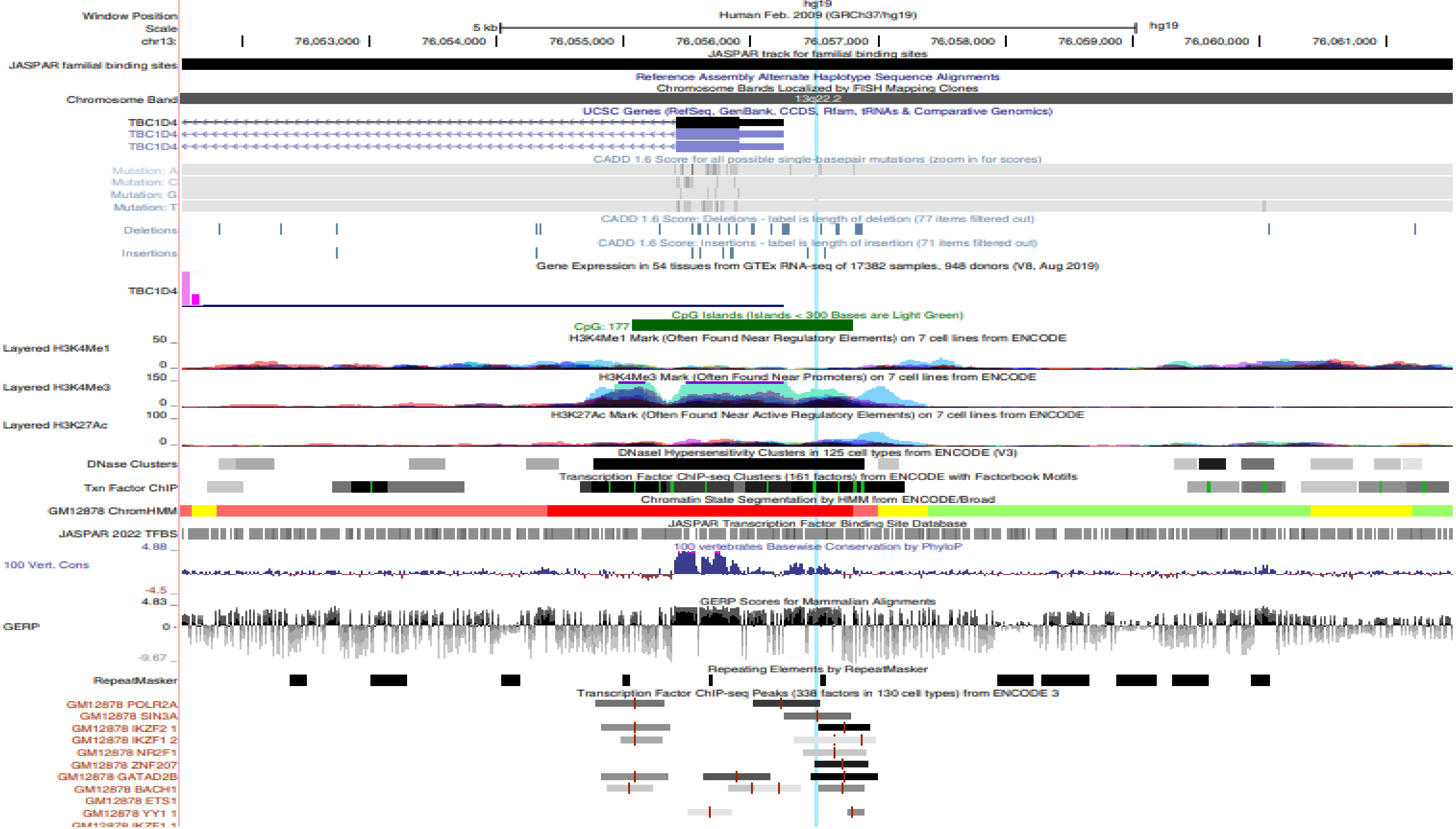

Supplementary Figure S8. *TBC1D4*: 13_76056522_G_A

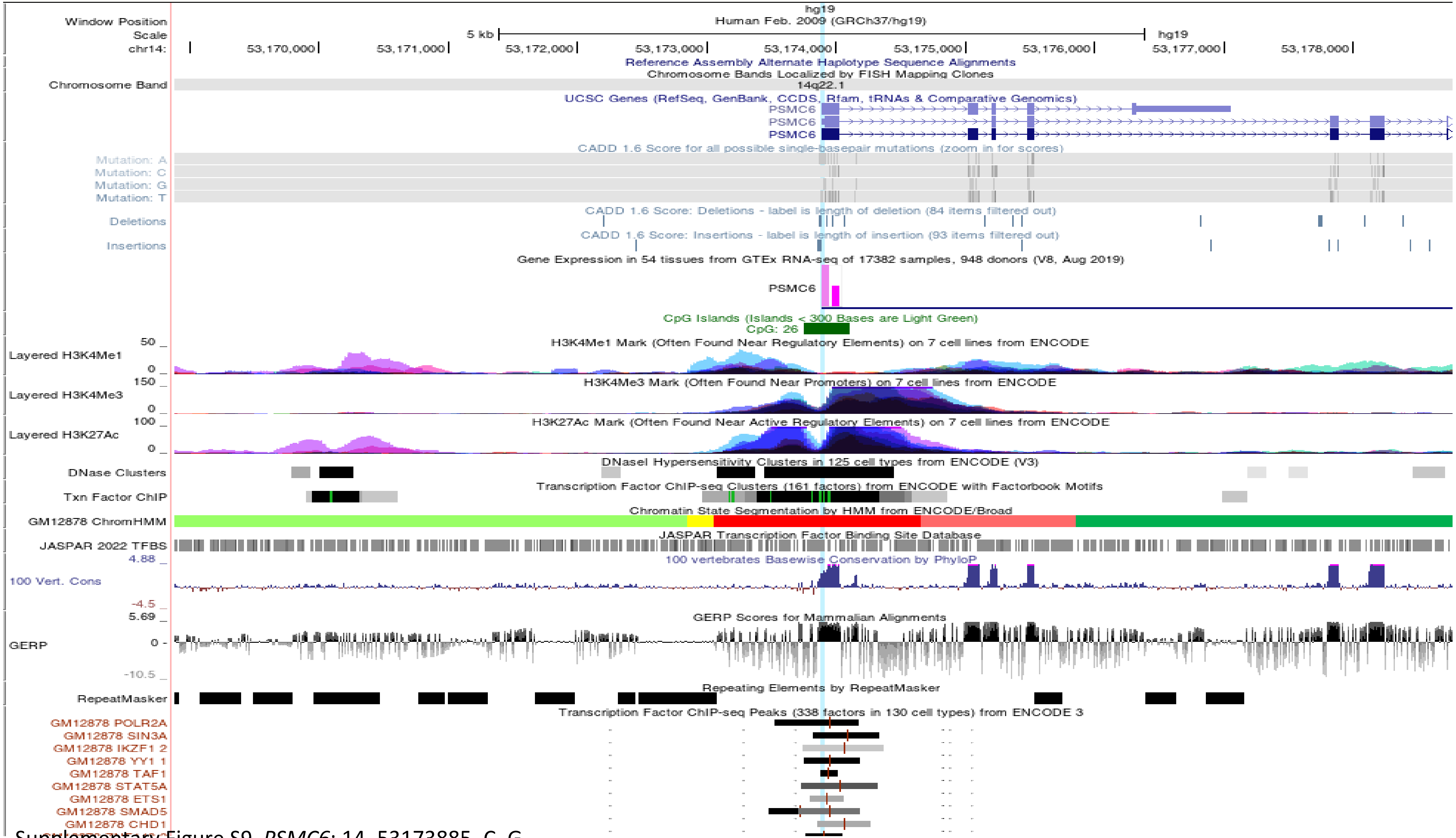

Supplementary Figure S9. *PSMC6*: 14_53173885_C_G

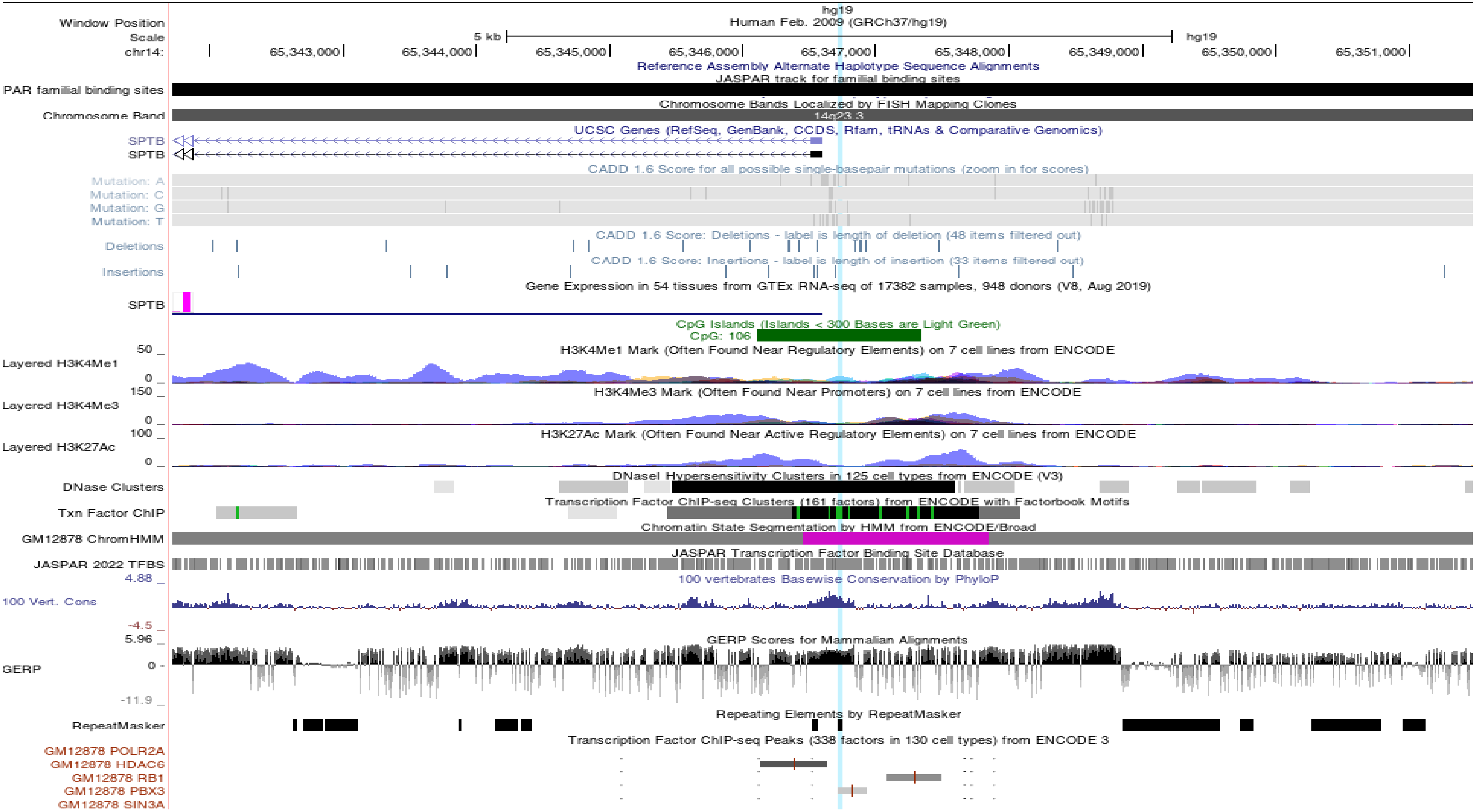

Supplementary Figure S10. *SPTB*: 14_65346721_C_A

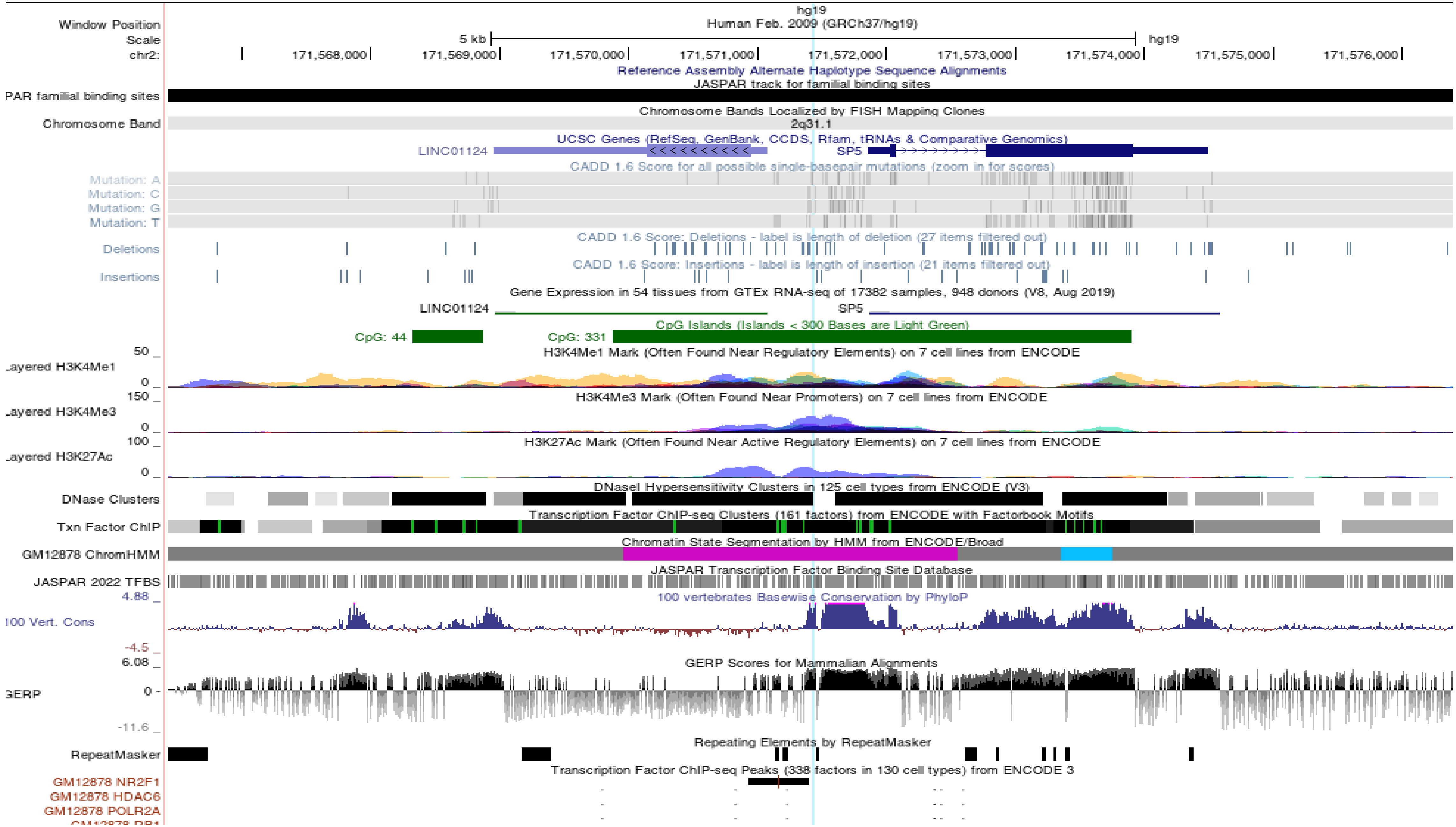

Supplementary Figure S11. *SP5*: 2_171571426_G_A

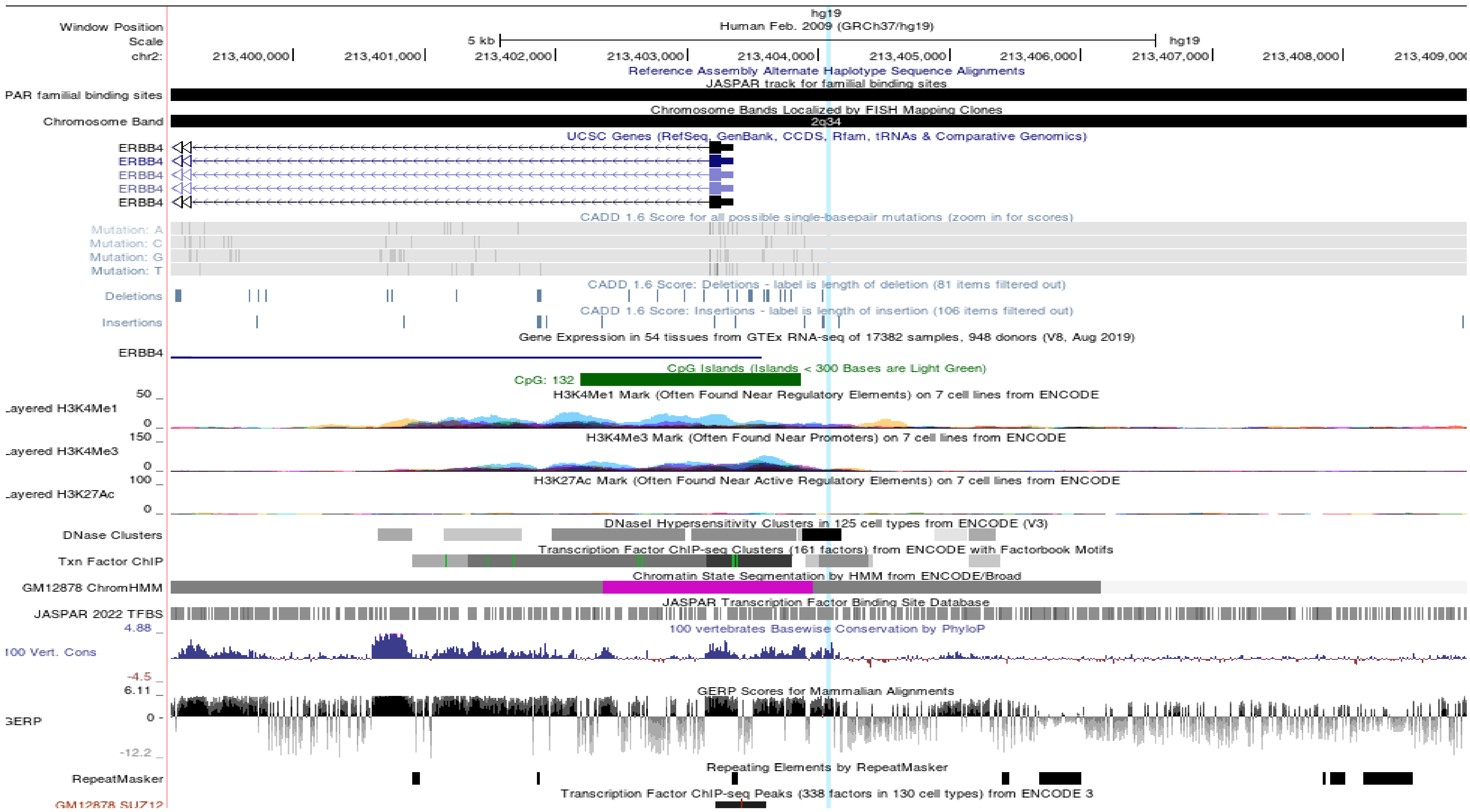

Supplementary Figure S12. *ERBB4*: 2_213404066_C_T

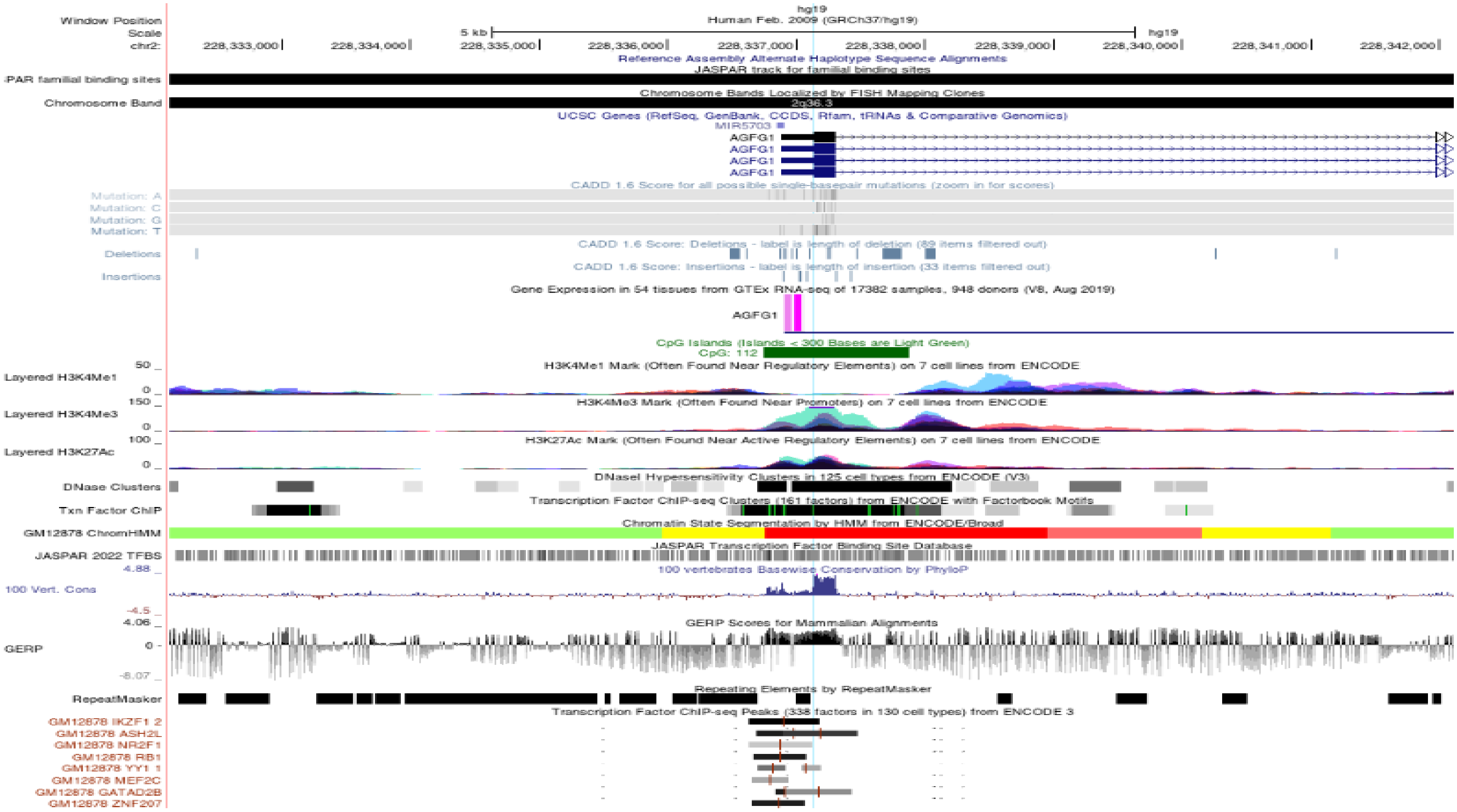

Supplementary Figure S13. *AGFG1*: 2_228337132_G_A

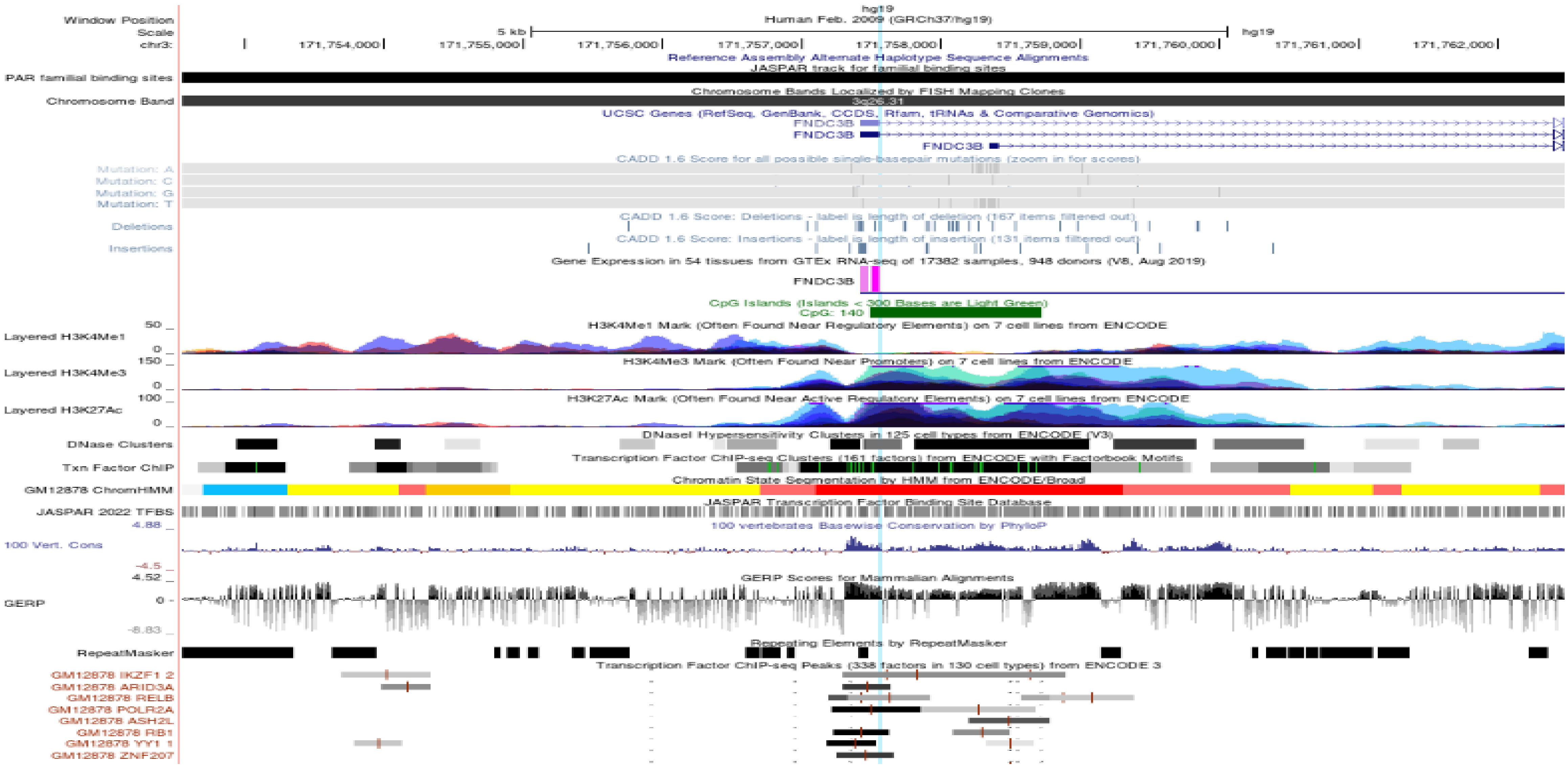

Supplementary Figure S14. *FNDC3B*: 3_171757553_C_A

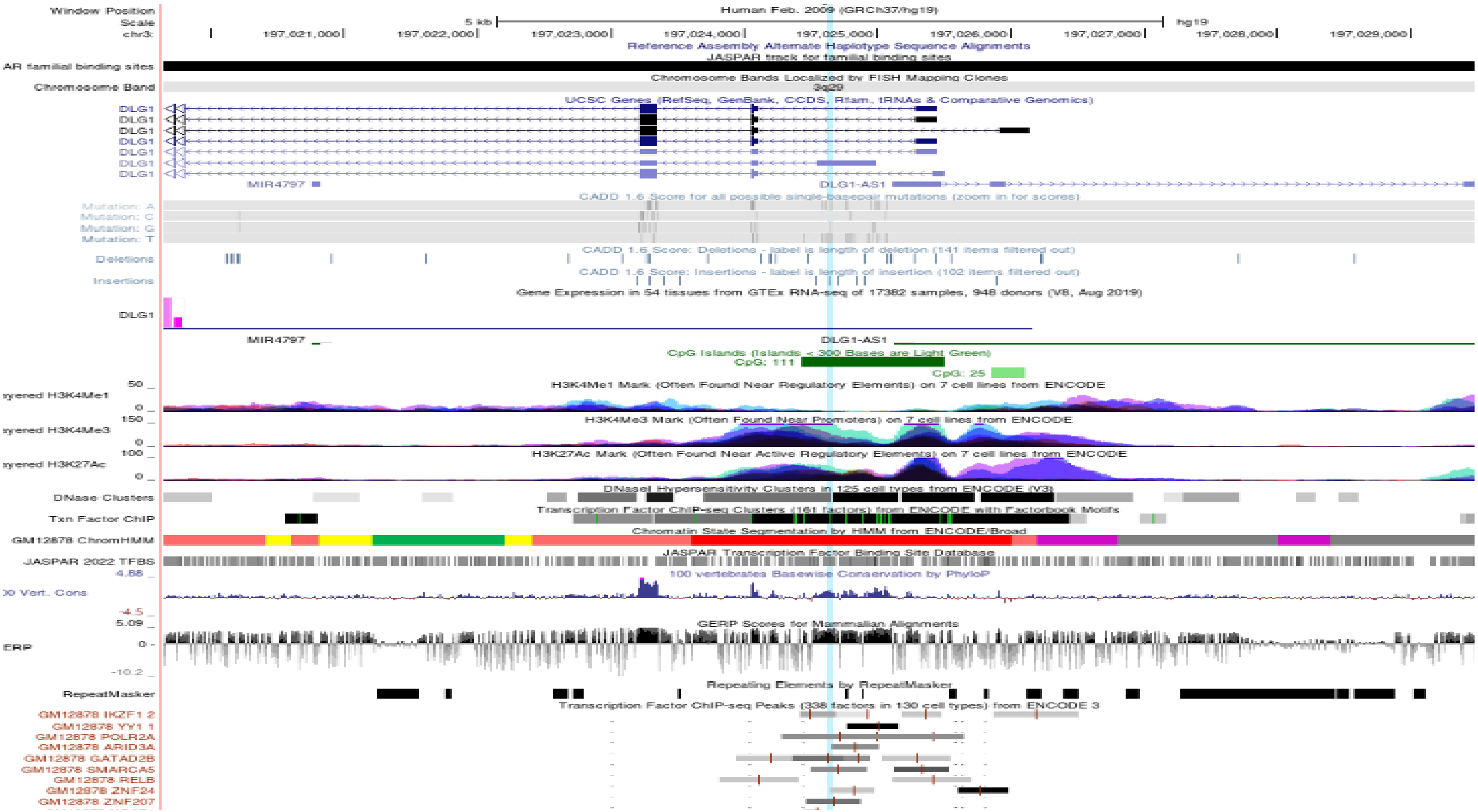

Supplementary Figure S15. *DLG1*: 3_197024641_C_T

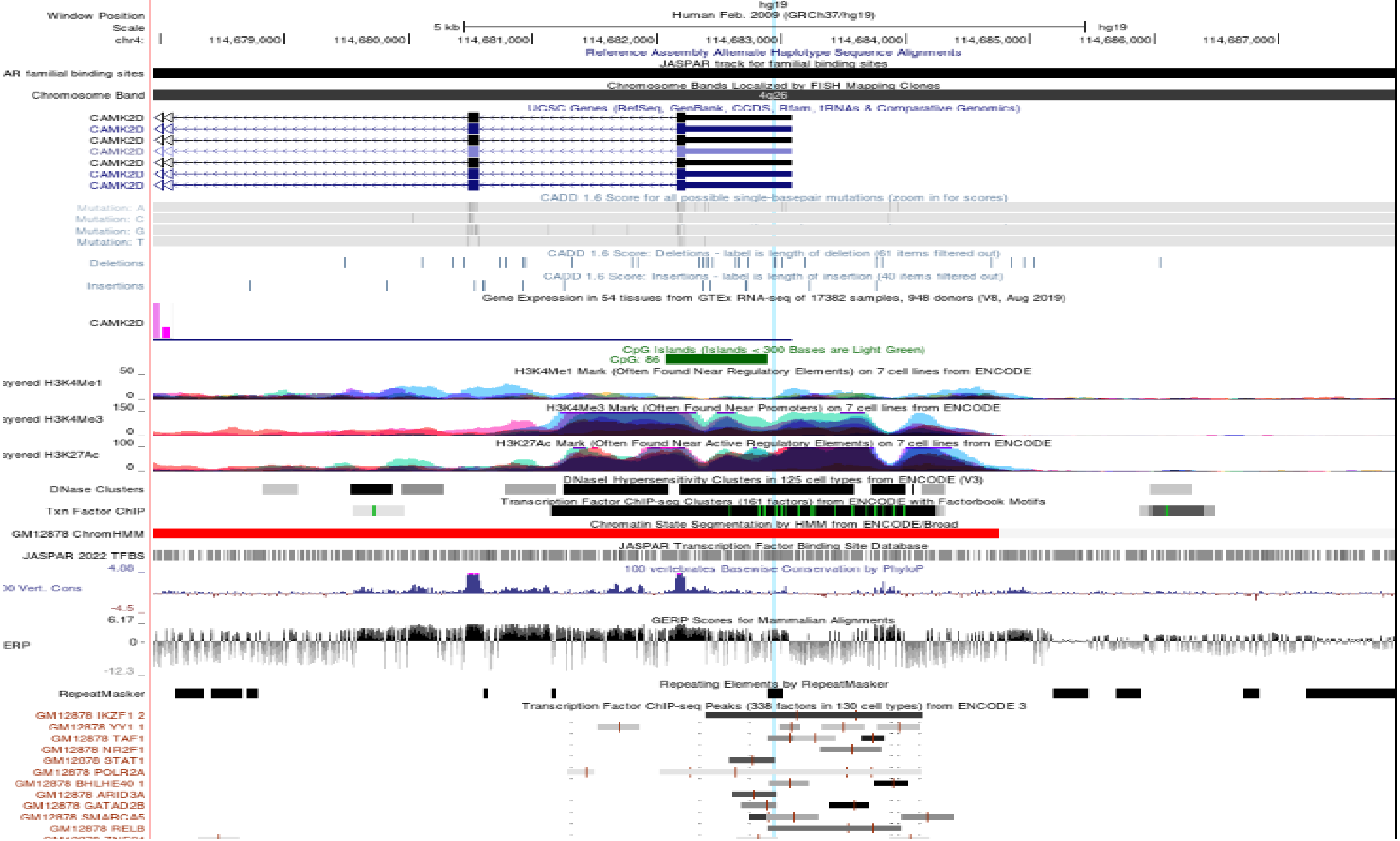

Supplementary Figure S16. *CAMK2D*: 4_114682943_TCCTCCTCCGGCG_T

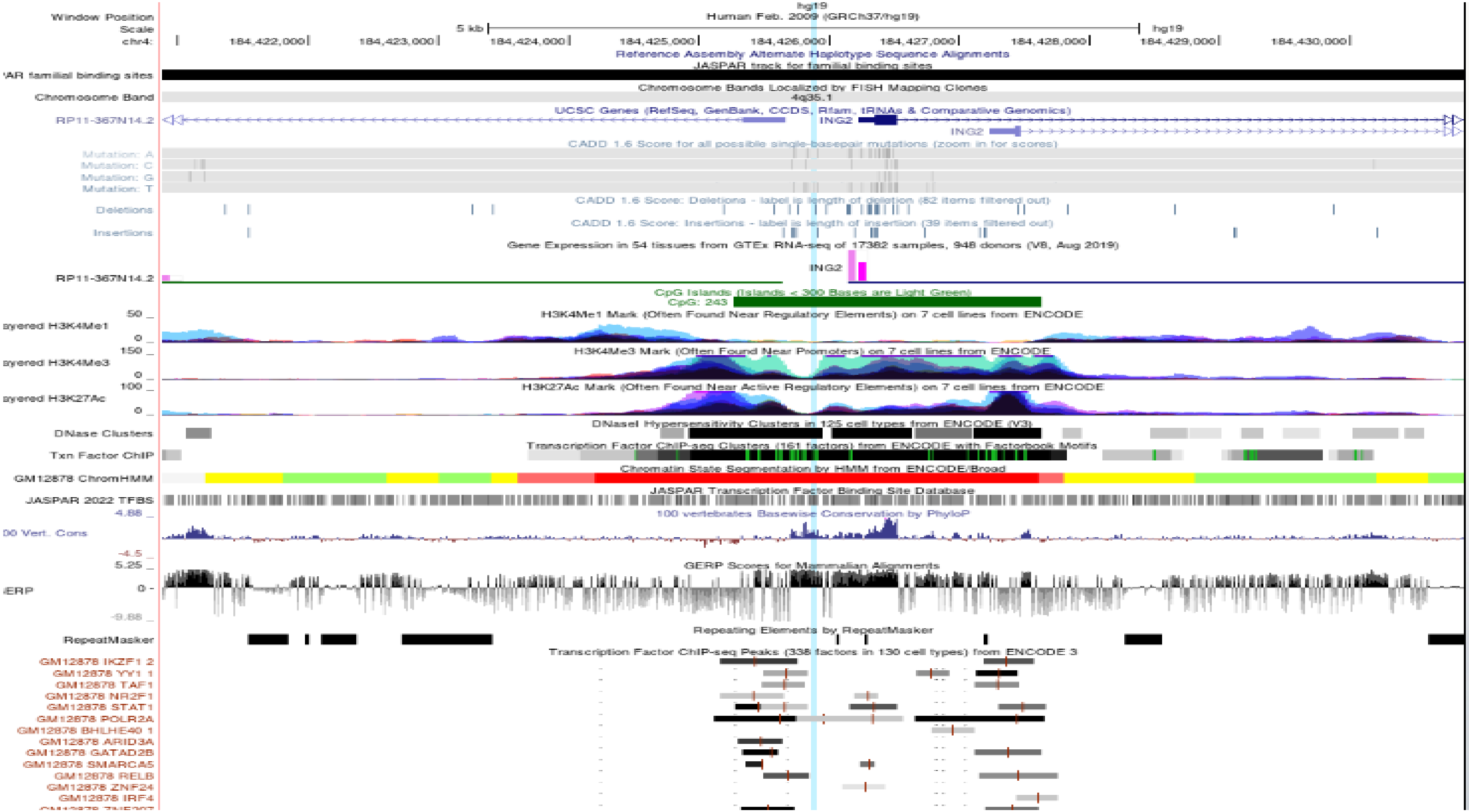

Supplementary Figure S17. *ING2*: 4_184425877_C_A

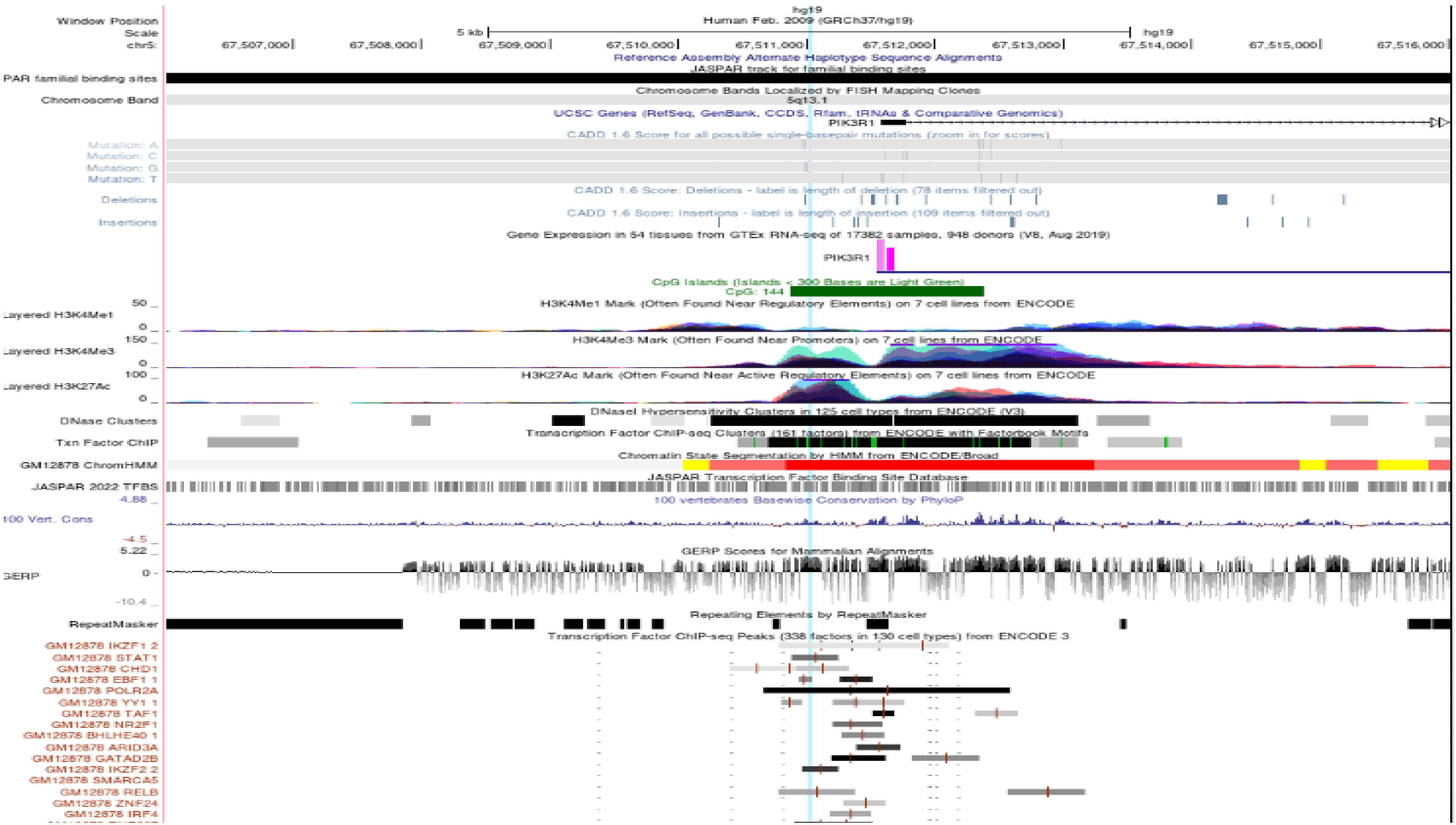

Supplementary Figure S18. *PIK3R1*: 5_67511017_G_C

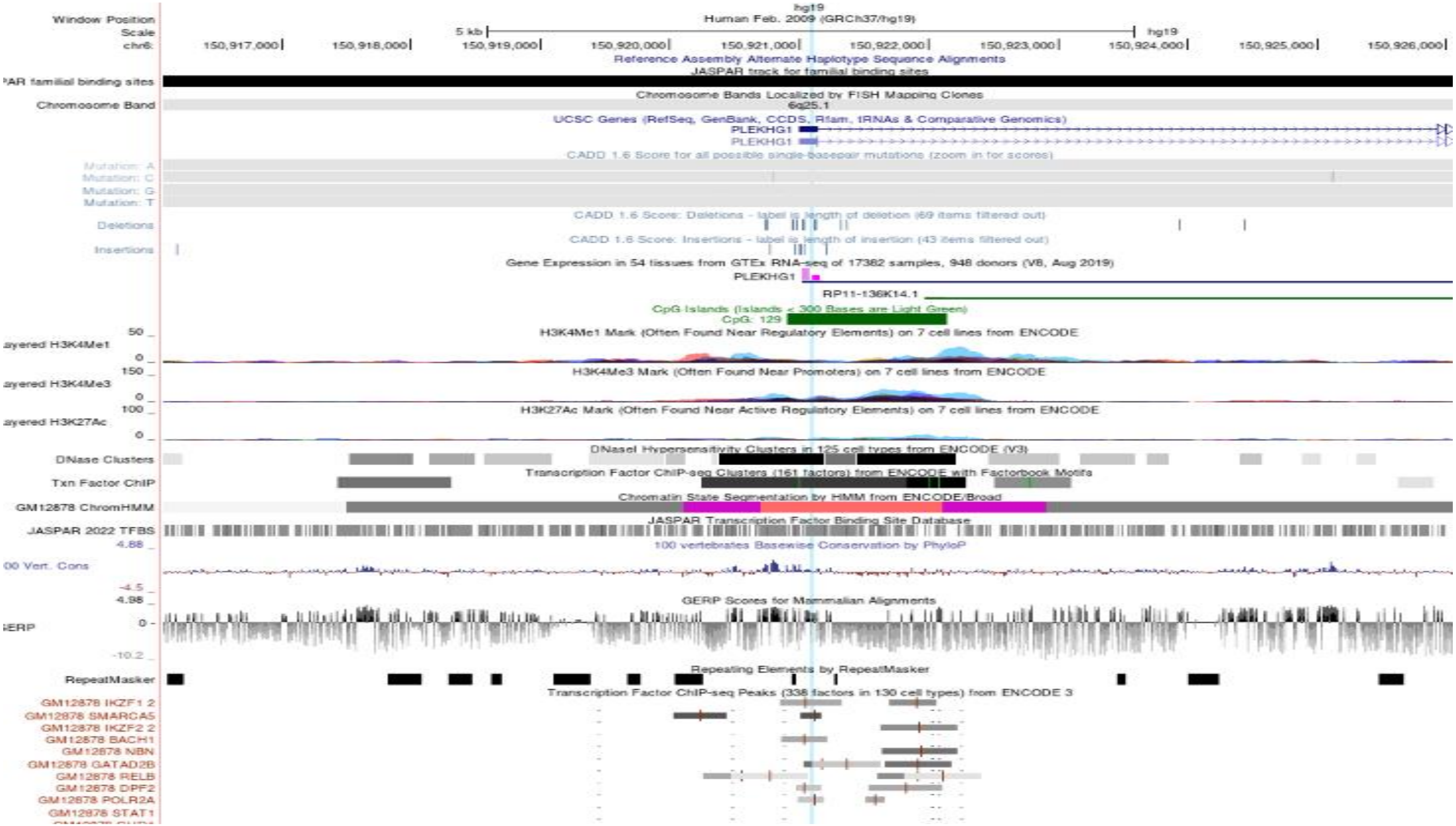

Supplementary Figure S19. *PLEKHG1*: 6_150921086_G_A

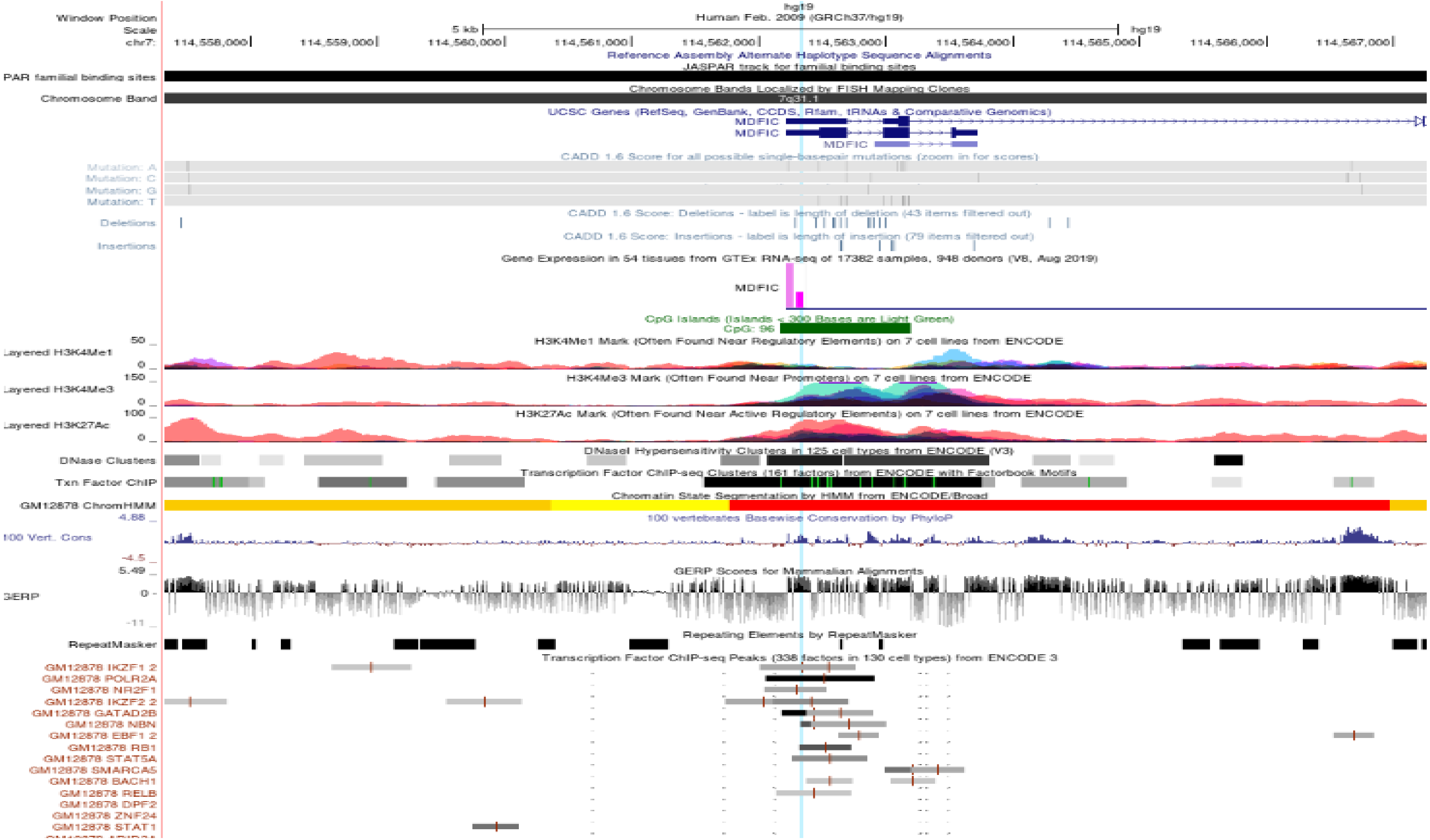

Supplementary Figure S20. *MDFIC*: 7_114562322_C_G

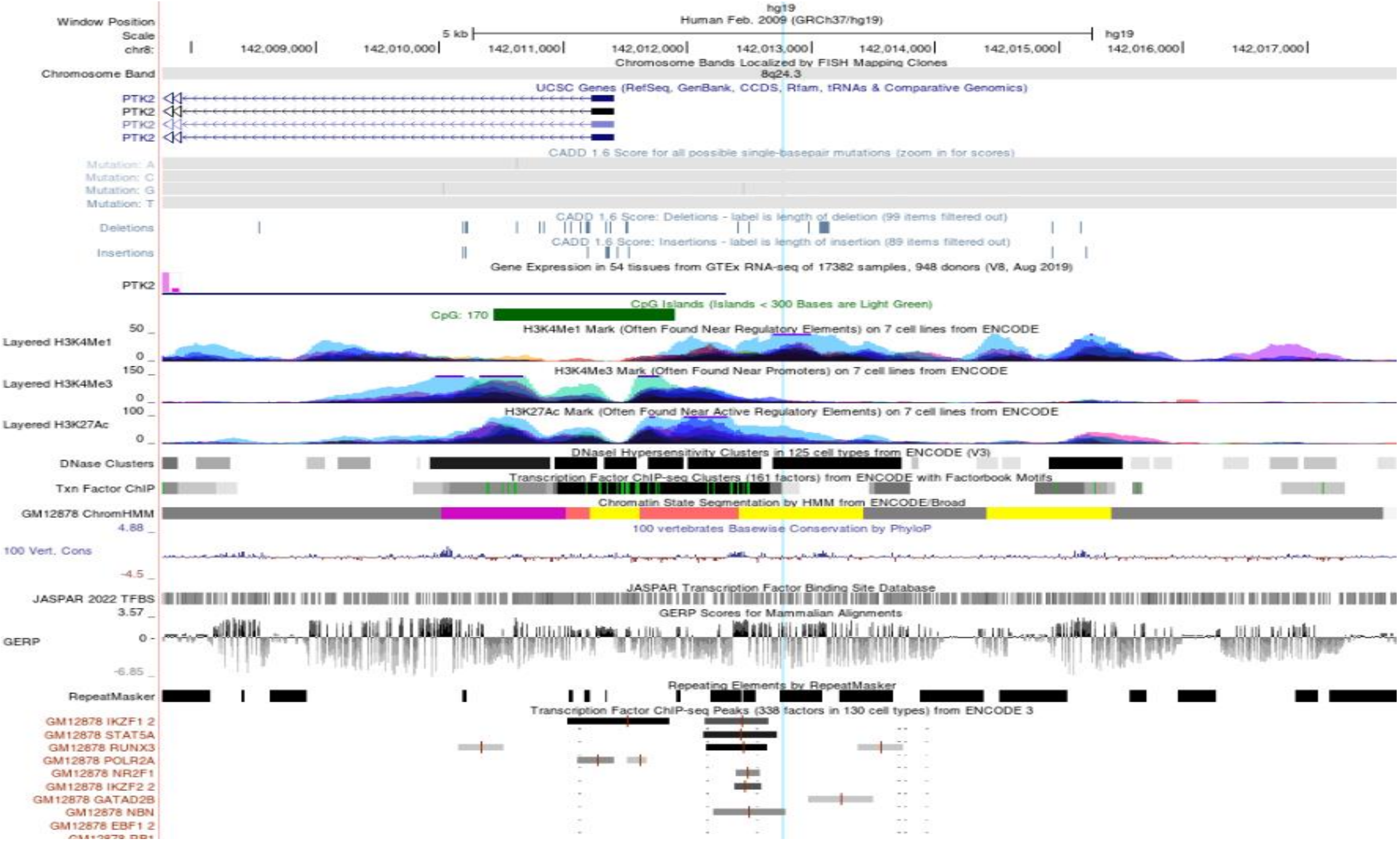

Supplementary Figure S21. *PTK2*: 8_142012766_C_T
